# An Address in the Atlanta Medical College, before the Æsculapian Society

**Published:** 1857-07

**Authors:** A. W. Griggs

**Affiliations:** Newnan, Georgia


ATLANTA

Medical and Surgical Journal.

VOL. II.] JULY, 1857. [NO. 11

### ORIGINAL COMMUNICATIONS.

### ARTICLE I.

*An Address delivered in the Atlanta Medical College before the Æsculapian Society.* By A. W. GRIGGS, M. D., of Newnan, Georgia, June 18th, 1857.

GENTLEMEN OF THE ÆSCULAPIAN SOCIETY:—We are proud of the distinguished honor of appearing before you on the present occasion. We are truly happy to witness such demonstrations of interest, manifested by this enlightened body, in subjects which have such prominence in our affections. We are only sorry, that we had not have had more time to have devoted to preparation, in order that we might have at least met your expectations of an address. This evening by association, the pleasant reminiscences of the past, cluster in golden splendor before the enraptured vision. Joy first diffuses her chameleon hues to the delighted spirit, but in a moment more we find ourselves lost in the midst of most melancholy reverie. The fond recollection of friends with whom we were once accustomed to meet in conventions like this, excites us as by a sudden shock from an electrical machine,—but the sad thought, that we will meet with but few of them ever again in life, produces an irresistible depression. But we will not pause here, to indulge such gloomy feelings. For the future claims greater respect than the past. Hereafter is of greater moment to us than heretofore. The sands of life are fast running—the last dust will soon have fallen and we be called to give an account of our stewardship on earth.

Shall we bury our talent in the sordid clay, or shall we present it to our Lord, both principal and interest? Shall we gaze upon the world in its busy commotion, and pass our precious moments in unprofitable engagements? We must labor. Idleness is incompatible with the duties of both students and practitioners of medicine. So much is to be acquired, that ages upon ages might roll on, and pour their plenteous accumulation into the mighty ocean of knowledge, and yet mysteries would be unexplained, and the most beautiful phenomena would take place in our midst unperceived. So vast is the field of our operation, that hundreds of years will be insufficient time, for the perfect elucidation of many important theories and subjects of medical science. How unreasonable, then, is indolence, when our task is so tremendously great. Among the young and the gay, science generally finds but few admirers—but *here* we are happy to find that many are striving to enter her pearly streets, and stand as watchmen on the heights of her towers. When our eyes are made to behold, here in the rich halls of medical learning, even in our own beloved Atlanta, so many young champions enlisted for life, to do battle in the great cause of philanthropy, new impulses arise, and new energies are created, and thus we are impelled in a direction upward and onward, by the effect of two great forces,—ambition for the success of the medical profession, and a desire to relieve the sufferings of our race everywhere. This, gentlemen, is the place where you can make every preparation to fit you for the mighty theatre of future action; it matters not whether you be called to administer as physicians or to operate as surgeons. You are presented with no inferior advantages—medical science is taught here in all its purity and elegance; your professors are more anxious to instruct you in correct principles, and have you understand them thoroughly, than to gratify any selfish ambition in warping your minds to suit their own peculiar views and opinions; they tender you all the recent improvements in medicine and in surgery. It is yours now to walk in the green pastures of science, and drink the sweet waters of her ever bubbling fountains. You are surrounded on every side with examples of energy and enterprise. You breathe the very atmosphere of improvement itself. The wheels of civilization, are bearing you on with electric speed to the goal of perfection. As has been said, it is an age of improvement; "our seas are white with the sails of commerce," our railways and canals intersect the country in every direction. The humming of spindles and the ringing of anvils are to be heard in nearly every city in the Universe—whilst our rich mines of coal and of copper are furnishing thousands of laborers with employment—by which they support themselves and their families. Improvement after improvement, and discovery after discovery, are constantly being made in the arts and sciences. The expansive power of steam in propelling machinery to which it has been applied, has redounded no less to the cause of education than to the financial interests of the country. Once we were albut strangers in our own land; but with the present facilities for traveling, with comparatively small expenditures of money, we can acquaint ourselves with different sections of both the new and the old world. To-day we may walk in the verdant fallow grounds of the sunny South, and to-morrow, we may nearly reach the frozen lakes of the North. The magneto-electric Telegraph conveying dispatches over land and under ocean, has filled the world with wonder and surprise. The time is at hand when we shall be able to know in a few hours, what is transpiring in the most distant parts of the earth. The electric wires will encircle the terrestrial globe, and then the expeditious communication of news will be easy to every land and every people. Fraternal international intercourse, will be encouraged and established everywhere. Misunderstandings will the more rarely occur, will be the more easily explained, and the more satisfactorily adjusted. But whilst so great improvements are taking place in the rest of the world, what are medicine and surgery doing for themselves? are they lagging behind in the distance? are they relapsing into superstition and idolatry? Have they become unmindful of the high destiny to which they belong. Have they stopped, outstripped by the rest of the world, and given up the chase in despair? No, no! is reverberated from earth to heaven, and the echo fills the immensity of space with its swelling sound. They rise in grander magnificence, and in more lovely proportions than the sun ever glowing in the celestial vault. They shine with more glittering splendor, than the beautiful colors of the Kaleidescope. Their harmony is more agreeable than could have been the sweet strains of Orpheus' music, when he played at the gates and unlocked the dungeons of hell. Corydon may sing of the beauty of Alexis, and the voluptuous charms of Arethusa may excite the sensual mind; but medicine, clad in her beauteous raiment, will attract us to her bosom forever. She proudly points to her rich trophies, and tells us that they are ours. The star of her glory has come from the east, and to-day is twinkling in the Southern sky. The lofty spires of her temple, pierce the towering clouds of adversity and scatter them broken to the wind. She is pure and lovely where e'er you find her home, whether in the ancient cities of the east or the vine clad bowers of the Floridas. Gentlemen have you ever thought of the superior claims of the medical profession. In my humble opinion, the physician ought to occupy the highest position in the estimation of the people. The gifted son of science, he sacrifices every personal pleasure and every bodily comfort, and ministers to the continual wants of the sick and the afflicted. When he would delight himself in the society of his friends and family, he is called away—in the midst of his conversation. When the Holy sabbath comes and all others lay aside their daily business, he is often not permitted to listen, unmolested for an hour, to the preaching of the gospel of salvation. "Oft in the stilly night," when her sombre curtains veil the world in darkness, "Hallo! hallo!" echoes through his chamber, and he is aroused from sweet sleep, rendered more refreshing by the fatiguing labors of the day. He is exposed to every vicissitude of heat and of cold. Nothing excuses him from duty so long as he is able to ride. Slight circumstances excuse the votaries of other professions, and the people do not think to complain. A thousand perishing souls may be assembled awaiting the coming of a minister to expound the everlasting truthes of the bible, and enlighten their benighted minds; but an inclement day, or a sick family would be deemed a sufficient excuse for his non-attendance. The rain may fall in torrents—the tempest may prostrate the forest in wild fury—the lightning's vivid flash may play upon the bosom of the angry storm, but the philanthropic physician, out-braving the dangers of the conflicting elements, mounts his accustomed horse, and visits patients, who, poverty stricken, can never remunerate him for his services—and yet these voluntary acts of kindness are too often considered matters of compulsion. In this the world is vastly, yea, most egregiously mistaken. The physician, no more than the counsellor at law, is compelled to perform gratuitous service. He is an independent character, and belongs to no one but himself and the profession. Think, for a moment, what would or could the world do without his labor and his knowledge. It is true that sometimes in the absence of epidemics, when the country is comparatively healthy, men forget the past, and say that he is of but little advantage to the people; but when disease begins its ravages in the land, they are the first to employ, and offer their whole estate for relief and restoration. The life of the physician is a life of vexation and trial. He is subject to every imaginable difficulty; being a public man, and surrounded by dishonorable competition, he often feels the blasting, withering breath of slander, threatening his immediate destruction. The sons of illegitimate medicine, have no more effectual way of rising to *notice* successfully, than by misrepresenting the policy of others. If you oppose the introduction of their quack remedies in the families of your patrons, they charge you with prejudice. To them, gentlemen, I have sworn eternal hostility—I have opened a war of extermination with them, which shall never abate nor subside, so long as a patent pill, powder, or preparation of the kind, can be found in our midst. I thank God, that most of my patrons hold them in as great contempt as I do myself—some time back our village was perfectly infested with these beasts of prey—our citizens have been humbugged so often by such boasting whelps, that the magic-working electric oil, would now go begging in the lowest classes. These fellows can be stopped. Are you not aware, that no person, according to the statutes of this State, can engage in the sale of medicines, unless he be a regular graduate, or licensed by a board of physicians, under the penalty of a fine of five hundred dollars and imprisonment, I believe, in the county jail for the term of three months, for the first offence.

The peddling of patent medicines ought to be a penitentiary crime; for it is by far worse than gambling at *farro*, or any other game at cards. Truth is mighty, and will prevail against them. Their days are numbered whenever the sons of Æsculapius turn their musketry among them. They will hunt them in their hiding places, and will find them as they skulk in their secret holes and retreats. Also, we have to conflict with the self-important Botanic, who discourses so eloquently about Lobelia and Capsicum; the Homeopath, with his pills of oystershells and sugar, talking of infinitesimals; the hydropath, challenging the world, with a great noise about water.

Now, we are satisfied, that if any philosophy exists in the doctrines of these systems, as they are called, it finds its origin in ours. Their advocates are confined to the use of a few remedies, whilst we, gentlemen, make all things which legitimately and honorably belong to medicine, subservient to our purposes, in the relief of pain, and the eradication of disease. We exclude no remedy which properly belongs to the materia medica. Why should we be prejudiced against a legal remedy: all we want, is to restore our patient to health. We had as soon that lobelia should cure as calomel. We had sooner that water should cure than any other remedy in the wide world; but they will not do it. Gentlemen, meet these things fearlessly and boldly. A man of science has but little to dread from a collision with such contemptible quacks, in an enlightened community. Your office is high—your responsibilities great. Conduct yourselves with such propriety as becomes your noble profession. You will soon have to act your parts in the grand drama of life. The janitor's bell will soon call you no more to these delightful seats of the sanctuary. Then it will be yours to smooth the rugged pathway to the grave; it will be yours to give consolation to the afflicted in body and in spirit, whether they dwell in the carpeted halls of the rich, or in the miserable hovels of the poor. Never forget your position as friend, benefactor, and physician. Ever be kind, amiable, candid, accommodating, and attentive. Strive for the elevation of your profession, and for the maintainance of its honor pure and inviolate; defend its claims with zeal, and study its rising interest as your interest, and as the only safeguard to your future position and eminence in the world.

